# Mapping Bubble Formation and Coalescence in a Tubular Cross-Flow Membrane Foaming System

**DOI:** 10.3390/membranes11090710

**Published:** 2021-09-15

**Authors:** Boxin Deng, Tessa Neef, Karin Schroën, Jolet de Ruiter

**Affiliations:** Food Process Engineering Group, Department of Agrotechnology & Food Science, Wageningen University, Bornse Weilanden 9, 6708 WG Wageningen, The Netherlands; boxin.deng@wur.nl (B.D.); tessa.neef@wur.nl (T.N.); karin.schroen@wur.nl (K.S.)

**Keywords:** cross-flow membrane foaming (CFMF), whey protein, (sub)millisecond, bubble formation, bubble coalescence, convective transport

## Abstract

Membrane foaming is a promising alternative to conventional foaming methods to produce uniform bubbles. In this study, we provide a fundamental study of a cross-flow membrane foaming (CFMF) system to understand and control bubble formation for various process conditions and fluid properties. Observations with high spatial and temporal resolution allowed us to study bubble formation and bubble coalescence processes simultaneously. Bubble formation time and the snap-off bubble size ($D_{0}$) were primarily controlled by the continuous phase flow rate ($Q_{c}$); they decreased as $Q_{c}$ increased, from 1.64 to 0.13 ms and from 125 to 49 µm. Coalescence resulted in an increase in bubble size ($D_{coal}>D_{0}$), which can be strongly reduced by increasing either continuous phase viscosity or protein concentration—factors that only slightly influence $D_{0}$. Particularly, in a 2.5 wt % whey protein system, coalescence could be suppressed with a coefficient of variation below 20%. The stabilizing effect is ascribed to the convective transport of proteins and the intersection of timescales (i.e., μs to ms) of bubble formation and protein adsorption. Our study provides insights into the membrane foaming process at relevant (micro-) length and time scales and paves the way for its further development and application.

## 1. Introduction

Foams are widely used in our daily life such as in pharmaceuticals, cosmetics and foods. Each application has specific demands for stability and functionality of the foams, and thus targets different bubble properties (i.e., bubble size and bubble size distribution), which are strongly influenced by the foaming techniques. Foams are conventionally produced and studied in high speed stirrer/mixer [1] and rotor-stator systems [2] that operate based on continuous fragmentation of larger bubbles into small bubbles. These traditional techniques are relatively easy to scale-up, but are also associated with limitations, including high energy input and limited control over the bubble properties. High shear and high surfactant concentration are needed to obtain a foam with relatively monodisperse and small bubbles. Alternatively, other methods like packed-bed system [3], microfluidic devices [4], and membrane systems [5,6,7,8] can directly generate individual bubbles with desired size and improved monodispersity. The packed bed and membrane systems are capable of achieving high throughput, while that is not the case yet for microfluidic devices that are still in need of up-scaling.

In a cross-flow membrane foaming (CFMF) system, the dispersed phase is injected into the continuous phase passing through a porous membrane matrix, and bubbles are detached at the membrane surface by the cross-flowing continuous phase. Compared to membrane emulsification, which has been substantially studied and reviewed [9,10,11,12,13], only few studies were conducted on membrane foaming and in most of these cases a high surfactant concentration (e.g., 10 wt % whey protein) was used and any short-term destabilization process (in particular, coalescence) was thus not taken into account. The bubble properties and resulting foam stability were evaluated at the outlet of the foaming systems and were then explained in terms of the applied process parameters and/or the membrane properties such as pore size [5,8]. However, these final bubble properties are an equilibrium state following the formation and re-coalescence of bubbles flowing in the continuous phase, with the latter process causing an increased bubble size and higher polydispersity [14]. To make a monodisperse foam, choosing a membrane with uniform pores is not sufficient, as was found with a micro-engineered membrane for emulsification [15]. It is thus crucial to first understand the bubble production process within very short timescales, and thereafter manipulate other factors accordingly, such as the surfactant concentration to stabilize the freshly-generated bubbles. To the best of our knowledge, such a fundamental study of bubble formation in a cross-flowing membrane foaming system is still missing. 

The goal of this study is to investigate the cross-flow membrane foaming process by in-line high-speed visualization of bubble formation and mapping of potential bubble coalescence. Whey protein isolate, which is often used in commercial food foams, is used as the surfactant, and we study the bubble properties such as bubble size (distribution) and formation frequency under the effects of transmembrane pressure, continuous phase flow rate and viscosity, as well as protein concentration. To indicate the occurrence of coalescence (if any), we observe the bubbles at two distinct positions in the foaming system: first, bubbles that are initially formed at the membrane surface, and second, bubbles flowing through an observation chamber at a short distance downstream from the membrane. 

## 2. Materials and Methods

### 2.1. Materials

In a cross-flow membrane foaming (CFMF) system, air bubble formation was studied in aqueous (Milli-Q, Merck Millipore) solutions of whey protein isolate (BiPro, 97.5% purity, Agropur, Granby, Canada) at various concentrations and viscosities. Viscosity was adapted using glycerol (99.5% purity, VMR International, Leuven, Belgium). The viscosity was measured at 20 °C in triplicate using an Anton Paar Rheometer (MCR301, Anton Paar GmbH, Graz, Austria) which is equipped with a Couette cell (C-DG26).

**Figure 1:**
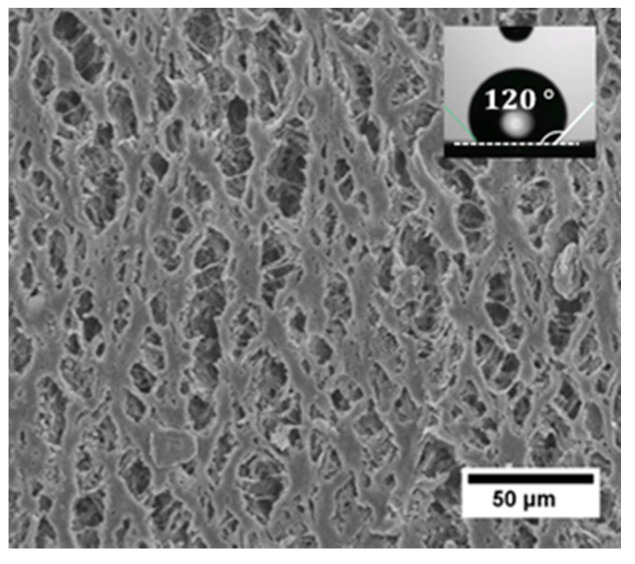
SEM photograph of the outer membrane surface. A snapshot for the macroscopic contact angle measurement is shown on top of the SEM image. The measurement is performed on a piece of unused membrane and the measured contact angle is about 120°.

A tubular-type polypropylene membrane with a macroscopic water contact angle of 120° was used in the CFMF system. The membrane has outer diameter of 2.7 mm and length of approximately 20 mm. The outer surface of the membrane shows a partially-interconnected pore network, with pore sizes ranging from a few to tens of micrometers (Figure 1).

### 2.2. Set-up

**Figure 2:**
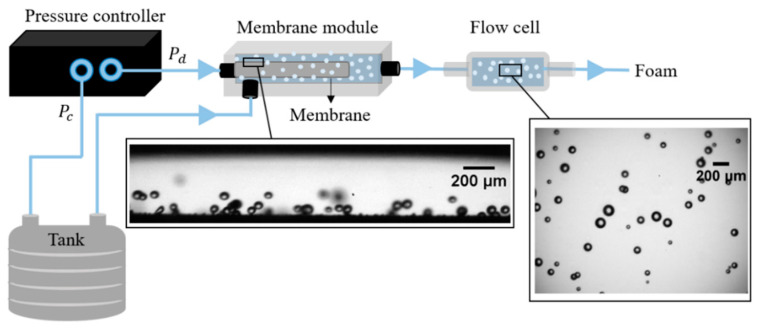
Schematic overview of the experimental set-up. The images show bubble formation at the membrane surface across from the continuous phase inlet and bubbles in the flow cell. The schematic is not drawn to scale.

The set-up for the CFMF experiment is schematically shown in Figure 2. The core of the set-up is the membrane module (that holds a membrane) where foaming takes place. The membrane module has two inlets for the air and continuous phase, a tubular chamber of 3.8 mm inner diameter in which foaming takes place, and one joint outlet. The membrane was inserted in the middle of the module, and a pressure controller fed air and continuous phase (via a feed tank) to the module. Between the inner wall of the module and the outer surface of the membrane, there is a gap of 0.55 mm. Bubble formation takes place by the action of the cross-flowing continuous phase that shears off the bubbles from the membrane surface, and next transports them towards the exit. 

Bubble formation can be observed along the membrane (see Figure 2). We chose a position near the continuous phase inlet albeit on the opposite side, marked by a rectangle in Figure 2. Additionally, a quartz flow cell (Type 55, Fireflysci, Ottawa, Canada) was connected to observe bubbles at 10 or 100 cm downstream of the membrane module, while keeping the total tubing length (and the flow resistance) constant. The flow cell has a height of about 0.4 mm, which allows us to observe undeformed bubbles in a more-or-less single flow plane. To investigate the bubble properties over time, we analyzed bubbles formed upstream across from the continuous phase inlet and compared them with bubbles flowing downstream through the flow cell (see insets of Figure 2).

### 2.3. Membrane Foaming

The flows of the air and continuous phases were both driven by pressure and controlled with the digital pressure controller that is operated with Smart Interface Software (Elveflow^®^, Paris, France). First, the (dispersed) air phase was injected into the system at pressure $P_{d}$. The pressure drop over the air phase occurs mainly across the relatively narrow pores of the membrane, such that the air pressure inside the membrane is also $P_{d}$. The continuous phase was then fed from the feed tank at pressure $P_{c}$, which drops more gradually over the full flow path, from $P_{c}$ at the flow controller to zero (absolute pressure) at the outlet. We estimated the flow resistance of each system component and found that at the continuous phase inlet of the membrane module, the pressure $P_{c}$ has dropped by approximately 55%; and the effective continuous phase pressure is thus defined as $P_{c,eff}$ that equals $P_{c,eff}=P_{c}\times \left(1-0.55\right)$. From this, the transmembrane pressure $P_{trm}$ is calculated by $P_{trm}=P_{d}-P_{c,eff}$. The pressure $P_{trm}$ is the effective pressure drop over the pores and drives the bubble formation. 

The bubble formation was investigated under the effects of process conditions and fluid properties. Firstly, the bubble formation was studied as a function of $P_{trm}$ in the range of 100–400 mbar. The corresponding air flow rate ( $Q_{d}$) was estimated from the volumetric production rate of foam collected at the outlet, with the liquid entrapped within the foam being subtracted. A constant continuous phase flow rate ($Q_{c}$) was applied to eliminate potential differences in convective transport of proteins towards the air/water interface. Secondly, for a given $P_{trm}$, the bubble formation was evaluated under the effect of $Q_{c}$—the volumetric flow rate measured by weighing the outflow of the system. It varies between $6.4\times 10^{-6}\;m^{3}/s$ and $2.1\times 10^{-5}\;m^{3}/s$, which corresponds to a cross-flow velocity of 1.6–5.4 m/s in the membrane module. Our window of operation is determined by pore activation and possibility of visualization. Operation at low $P_{trm}$ or high $Q_{c}$ is limited by low activation of the pores. Operation at high $P_{trm}$ or low $Q_{c}$ is in principle possible and leads to high bubble productivity, but the bubble fraction is then too high for visualization. Lastly, to study the effect of continuous phase viscosity (η) and protein concentration (c), process conditions were fixed to $P_{trm}=$ 200 mbar and $Q_{c}=1.5\times 10^{-5}\;m^{3}/s$, using either constant c=1 wt % with η varying as 1.3, 1.5 and 2 mPa·s, or constant η = 1.3 mPa·s with c varying as 0.5, 1.0 and 2.5 wt %. 

For practical reasons, protein solutions were re-used (for up to 10 times) during the experiments. Since the reduction of protein concentration over time was under 20% (Figure S1), this effect was ignored. The module and tubing were cleaned with MilliQ-water between different experimental conditions, and the membrane was replaced once the pressure applied on the air phase was switched off and on again (every day). All the experiments were performed at ambient temperature.

### 2.4. Image Analysis

A high-speed camera (FASTCAM SA-Z, Photron Limited, Tokyo, Japan) is attached to the inverted microscope (Axiovert 200 MAT, Carl Zeiss B.V., Breda, The Netherlands) and used to observe and visualize bubbles in the CFMF system. Videos were recorded at 20,000 frames per second and with a resolution of 0.314 pixel/µm. For each experimental condition, two videos were recorded at two distinct positions in the CFMF system: capturing either bubble formation at the membrane surface across from the continuous phase inlet (Figure 2); or bubbles flowing through the flow cell during their transport to the outlet. 

A custom-written code in MATLAB R2018b was used to calculate the diameters of bubbles that are directly formed at the pore openings ($D_{0}$, averaged for up to 100 bubbles) and that are the result of coalescence ($D_{coal}$, averaged for up to 100 bubbles), respectively. Histogram plots of the bubble diameters were made to visualize the number-averaged bubble size distribution. Moreover, the bubble size distribution was also characterized by the coefficient of variation (CV), defined as CV=δ/D×100% (in which, δ the standard deviation and D the number-averaged bubble diameter). Average bubble formation frequencies were estimated by $f_{0}=Q_{d}/V_{0}$ and $f_{coal}=Q_{d}/V_{coal}$, where $V_{0}$ and $V_{coal}$ are the volumes corresponding to $D_{0}$ and $D_{coal}$, respectively. Furthermore, two timescales were estimated during a bubble formation cycle using the videos. The interval time is the lag time between two bubble growing processes at the same pore opening, and the bubble growing time is the time of actual bubble growth at a pore opening. These were measured for each experimental condition, and the values were averaged for up to 30 bubbles.

## 3. Results and Discussions

### 3.1. General Bubble Behavior in CFMF 

We study bubble formation as function of the transmembrane pressure ($P_{trm}$). Bubble formation only occurs if the transmembrane pressure exceeds the activation pressure. This activation pressure equals the capillary pressure of the meniscus in the narrow pore, which is defined as $P_{cap}=\frac{4\gamma \cos\left(\theta \right)}{d_{p}}$, where γ is the surface tension between the air and the continuous phase, $d_{p}$ is the diameter of the pore (opening), and θ is the contact angle between the continuous phase and the membrane surface [12]. At $P_{trm}$ as low as 50 mbar, a few pores form bubbles at an extremely low formation frequency (too low for quantitative analysis of $D_{0}$ and corresponding $f_{0}$). The activation pressure for bubble formation is thus assumed to be approximately 50 mbar. For contact angles >90°, the air phase would flow out of the pores automatically, and the growing bubble would possibly spread out at the (hydrophobic) membrane surface, which would lead to extensive coalescence at the membrane surface [11,16], which is not observed in the present study. Thus, the macroscopic contact angle (120° as measured for an unused membrane) is not the actual contact angle in the system. Possible explanations are differences between micro- and macroscopic contact angle, which is known to occur on porous surfaces such as membranes, but more probably protein adsorption renders the membrane surface more hydrophilic (<90°). 

**Figure 3:**
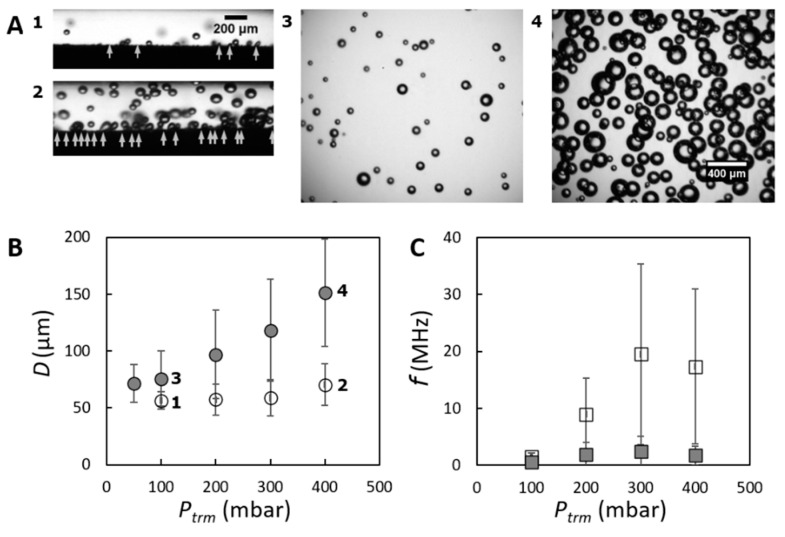
Bubble formation as function of transmembrane pressure, $P_{trm}$ (at constant $Q_{c}=1.5\times 10^{-5}\;m^{3}/s$ and c=1 wt %). (**A**) Snapshots of bubble behavior obtained when using different transmembrane pressures (low—1,3 and high—2,4): 1,2—visualization of bubble formation at the membrane surface in the module; 3,4—visualization of bubbles that are flowing through the flow cell (10 cm downstream). It is noted that in 1,2, only pores located at a single tangent line of (part of) the membrane are in our focal plane, while in 3,4 bubbles formed from the whole membrane surface (circumference and length) are present. In 1,2, the arrows roughly indicate the position of active pores. (**B**) Bubble sizes $D_{0}$ and $D_{coal}$, with a typical CV of ~20% and ~40%, respectively. (**C**) Bubble formation frequencies. The large error bars reflect the width of the size distribution, yet they are expected to be much smaller when studying individual pores. The bubble sizes (and the corresponding formation frequency) are indicated with two different symbols: $D_{0}$ ($f_{0}$ )—unfilled circle (square) and $D_{coal}$ ($f_{coal}$ )—filled circle (square).

We can estimate the effective diameter $d_{p}$ of (circular) pores that are activated based on $P_{cap}$= 50 mbar and γ varying between equilibrium value (~50 mN/m) and that of pure air/water interface (~72 mN/m). For θ we assumed a microscopic contact angle of about 80°. The estimated diameter of the activated pores is of the order of 10 μm, which corresponds with the largest observed pores. This means that at low $P_{trm}$, only the relatively large surface pores are activated to form bubbles, while the smaller pores need a higher activation pressure. When increasing $P_{trm}$ above 50 mbar, bubbles are increasingly present in the membrane module. This behavior is ascribed to the increasing number of active pores (Figure 3A1,2, indicated by the arrows) and the increasing frequency of the pores, with smaller pores having a lower frequency. 

Along the system, going from membrane module (where $D_{0}$ is estimated) to downstream in the flow cell (where $D_{coal}$ is derived), there is more substantial increase in bubble size at higher $P_{trm}$ (compare Figure 3A from 2 to 4 versus Figure 3A from 1 to 3). The two bubble diameters (estimated in the module and flow cell) and their corresponding formation frequencies are plotted to quantify the effects of $P_{trm}$ on bubble formation and stabilization (Figure 3B,C). The bubble diameter $D_{0}$ only slightly increases when $P_{trm}$ is increased to up to 300 mbar, showing a similar trend as that was reported in [8]; and it increases relatively more at $P_{trm}=400$ mbar. In contrast to the overall slight variation in $D_{0}$, the coalesced bubble diameter $D_{coal}$ significantly increases with $P_{trm}$. The difference indicates the co-existence of bubble formation and bubble coalescence in the 1 wt % whey protein system. $D_{coal}$ increases much more strongly than the rather constant $D_{0}$ for increasing $P_{trm}$ (Figure 3B), which is indicative of an increasing extent of bubble coalescence. Last but not least, with increasing $P_{trm}$ the frequency of coalesced bubbles was increasingly lower than the corresponding $f_{0}$ (Figure 3C). For a given continuous phase flow rate, bubble formation frequency is determined by both the $P_{trm}$ and the bubble size, which have opposite effects [17]. As shown in Figure 3C, $f_{0}$ first increases and then decreases with increasing $P_{trm}$. The decrease may be explained by the increased size of the bubbles, possibly in combination with more extensive coalescence (at high $P_{trm}$), and this may lead to the much lower overall $f_{coal}$. 

To confirm whether coalescence mainly occurs in the membrane module or continues downstream, we measured $D_{coal}$ at two distinct positions along the flow path, namely at a distance of 10 cm and further of 100 cm downstream the membrane module (Figure S2). The obtained $D_{coal}$ values are similar, which proves that bubbles coalesce mostly in the membrane module during or shortly after their formation, reaching a stable situation within a short term. This also allows us to limit ourselves to a flow cell position of 10 cm downstream for all the remaining experiments. 

### 3.2. Bubble Formation at the Membrane Surface

We first introduce the forces which dictate bubble formation. As was reported for cross-flow membrane emulsification systems, the bubble is subjected to four forces while growing at the membrane surface, which are the shear force imposed by the continuous phase flow rate and viscosity, the buoyancy force, the inertia force and the interfacial tension force [10,17]. For bubble formation in the present system, the interfacial tension force is the holding force, and scales as either $\frac{\gamma R_{p}^{2}}{R_{0}}$ or $\gamma R_{p}$, depending on the applicability of a force or torque balance ($R_{p}$ is the radius of the pore and $R_{0}$ is the in-line radius of bubble which grows towards $D_{0}$) [18]. The shear force, which scales as $v_{c}\eta R_{0}$, is considered to be the only driving force among the rest of the forces since the other two forces are at least two orders of magnitude smaller. The bubble stays attached to the pore opening and snaps off when, for a certain bubble size, the shear force exceeds the surface tension force [19,20]. The force or torque balance leads to $D_{0}\;\sim \;Ca^{-n}$, with $Ca=\frac{\eta v_{c}}{\gamma }$ the capillary number of the flow and power-law exponent n whose value is between 0.5 and 1. 

In our experiments, the continuous phase flow rate ($Q_{c}$), protein concentration (c) and continuous phase viscosity (η) are varied, and bubble formation at the membrane surface is studied. In accordance with the definition of forces, our experimental parameters can influence this force or torque balance: by increasing either the flow rate or the viscosity of the continuous phase, the shear force increases; by increasing the protein concentration, faster protein adsorption can lower the (dynamic) surface tension faster and thus rapidly reduce the holding force (that is if protein adsorption can appreciably take place within the very short timescales for bubble formation). In both cases, bubbles can be detached earlier from the pore openings [21,22]. 

**Figure 4:**
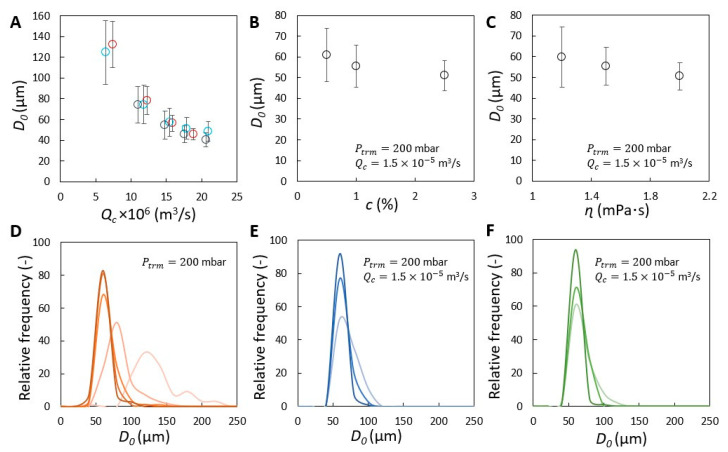
Properties of bubbles (with $D_{0}$) formed at the membrane surface. (**A**) Shear effect investigated for $P_{trm}$ equal to 100 (O), 200 (O) and 300 (O) mbar. Results at $P_{trm}=$ 400 mbar are not included, because bubbles that are initially formed at the pores cannot be measured due to bubble crowdedness. (**B**) Protein concentration effect. (**C**) Continuous phase viscosity effect. Corresponding $D_{0}$ values of figures (**A**–**C**) shown as size distribution obtained at $P_{trm}=$ 200 mbar are given in (**D**–**F**). Darker colors indicate the increase in the evaluated parameter.

The bubble size is firstly investigated as function of continuous phase flow rate. Please note that bubbles growing at the membrane surface tend to be deformed by the flow of the continuous phase, particularly when a high continuous phase flow rate ($Q_{c}$) is applied. $D_{0}$ significantly decreases as $Q_{c}$ increases (see Figure 4A), which is in line with what was reported for other membrane foaming systems [8,19] or membrane emulsification [23,24]. At lower $Q_{c}$, the bubble stays attached to the pore for a longer time, thus obtaining a larger size, while at higher $Q_{c}$, $D_{0}$ reduces to about 50 µm for the highest $Q_{c}$’s measured. The results agree with the proposed scaling with Ca with a fitted power-law exponent n= 0.8 ± 0.1.

$D_{0}$ almost shows no dependency on transmembrane pressure for $P_{trm}=$ 100–300 mbar for any given $Q_{c}$ (Figure 4A). At $P_{trm}=$ 400 mbar, the small increase in $D_{0}$ represents the volume of air added during the final detachment process of the bubble due to a higher air flow rate [24]. The strong dependency of $D_{0}$ on $Q_{c}$ indicates that the high shear flow dominates overall behavior; the effects of transmembrane pressure and fluid properties on bubble formation at the membrane surface can be neglected. 

Additionally, only a minor decrease can be observed in $D_{0}$ as function of protein concentration and continuous phase viscosity (see Figure 4B,C), which are both evaluated at $P_{trm}=$ 200 mbar and $Q_{c}=1.5\times 10^{-5}\;m^{3}/s$ (Figure 4A). By raising the continuous phase viscosity, the average bubble size $D_{0}$ is reduced to about 50 µm due to effects on increasing the shear force and potentially also the (dynamic) surface tension force. The decreasing trend with viscosity collapses onto the same $Ca^{-0.8}$ behavior (assuming a constant surface tension, Figure S3). For these experiments taken at larger Ca, the rather constant $D_{0}$ can be ascribed to the fast bubble detachment process induced by shear force exerted by the continuous phase [24]. When the protein concentration increases to 2.5 wt %, the $D_{0}$ decreases also just slightly to approximately 51 µm. The decrease is ascribed to faster protein adsorption which further lowers the (dynamic) surface tension and advances the force balance for bubble snap-off. Moreover, because the surface tension only can be decreased to a limited extent, bounded by the equilibrium surface tension of the air/water interface stabilized by whey proteins, the influence of protein adsorption on bubble size is moderate. Hence, bubble formation at the membrane surface falls into a regime where bubble snap-off is dominated by shear force, and under the conditions studied here, mainly controlled by the continuous phase flow rate [10]. 

The bubble size distribution varies with bubble size. The histogram plot indicates that bubbles with higher monodispersity are formed as $Q_{c}$ increases, with the main peak in the plot shifting from right to left (Figure 4D). In addition to the effects of $Q_{c}$, the bubble size distribution can further be narrowed by increasing the protein concentration and/or the continuous phase viscosity (Figure 4E,F). Bubbles with diameters in the range of 40–60 µm account for more than 90% of the size distributions (Figure 4E,F). The corresponding coefficient of variation (CV) is below 15% when the highest protein concentration and/or viscosity is used (Figure 4E,F). However, the average $D_{0}$ can be decreased only to a limited extent (to approximately 50 µm), with the smallest bubbles having a diameter of approximately 40 µm (corresponding to the left side of the histogram plot), which is independent of the experimental conditions; and the bubble size distribution cannot be infinitely narrowed. This can be ascribed to the characteristics of protein adsorption and the properties of the used membrane—the pore size and the pore size distribution. Specifically, when bubble formation is much faster than protein adsorption, the surface tension keeps constant as that of a pure interface, limited by the efficiency of protein adsorption; and moreover, the smallest pores that have a lower holding force, produce smaller bubbles thus widening the bubble size distribution [17], and determine the lower boundary of the bubble size. In addition, interactions between bubbles can influence the bubble size distribution by detaching the bubbles early on [25]. 

### 3.3. Bubble Coalescence 

To thoroughly understand the CFMF system, the bubble coalescence is also evaluated as a function of the same experimental parameters. Firstly, within the resolution of our experimental results, coalescence of bubbles growing at adjacent active pores is rarely visually observed. Instead, coalescence often happens upon collision amongst flowing bubbles or a collision between a flowing bubble and a forming bubble. To demonstrate the extent of bubble coalescence, we report here the bubble size measured in the flow cell ($D_{coal}$). In addition, we compare the volumes ($V_{0}$ and $V_{coal}$) of single and coalesced bubbles to estimate the number N of coalescence events a bubble has undergone: $N=V_{coal}/V_{0}-1$. Because $V_{0}$ may show some variation across different positions at the membrane surface (Figures S4-1 and S4-2), due to the pressure drop in the continuous phase and the presence of bubbles (which further influence the local flow rate and viscosity of the continuous phase), the absolute values of the estimates for N are shown in Figure S5, yet general conclusions can be drawn and will be discussed below.

**Figure 5:**
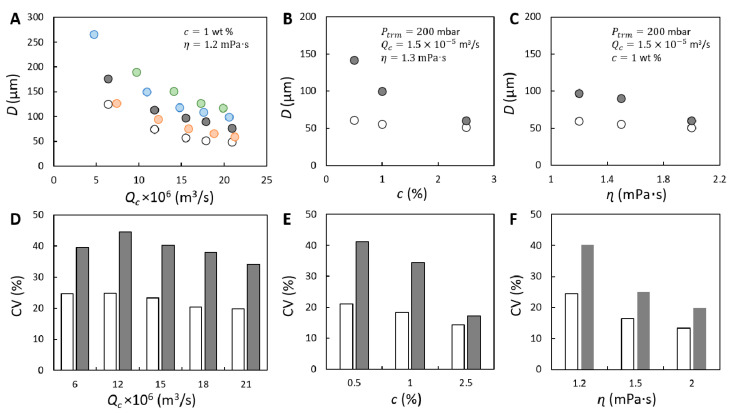
The size and size distribution of coalesced, stabilized bubbles. Bubble size ($D_{coal}$, presented with filled circle) under the effects of: (**A**) shear flow; (**B**) protein concentration; (**C**) continuous phase viscosity. In (**A**), the effect of shear flow on $D_{coal}$ is investigated for $P_{trm}$ equal to 100 (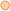), 200 (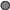), 300 (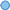) and 400 (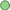) mbar. In (**A**–**C**), the size of initial bubbles ($D_{0}$ ) measured at $P_{trm}=$ 200 mbar is plotted for comparison and is presented with unfilled circle (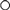). Coefficient of variation (CV) corresponding to $D_{0}$ and $D_{coal}$ measured at $P_{trm}=$ 200 mbar shown in (**A**–**C**) is presented in (**D**–**F**). The unfilled and filled bars represent the CV of initially formed and coalesced bubbles, respectively.

For a given transmembrane pressure, $D_{coal}$ decreases as $Q_{c}$ increases (Figure 5A) and is always larger than its corresponding $D_{0}$. Yet, N increases as a function of $Q_{c}$, which possibly resulted from faster bubble formation (i.e., less protein adsorption) (Figure S5). At any fixed $Q_{c}$, $D_{coal}$ as well as N increase with $P_{trm}$ due to increasing bubble crowdedness (Figure S5), and higher propensity to coalescence. Furthermore, as function of the protein concentration, $D_{coal}$ decreases strongly, much more strongly than $D_{0}$ (see Figure 5B). With the highest protein concentration tested (2.5 wt %), $D_{coal}$ is very similar to $D_{0}$, and the coalescence is effectively suppressed with N≈0 (Figures S5 and S6). When a sufficiently high protein concentration is used, more proteins are able to adsorb to the bubble interface before bubble–bubble interactions take place, thus preventing the bubble coalescence [26,27]. Lastly, $D_{coal}$ also decreases and converges to $D_{0}$ when a higher continuous phase viscosity is used (see Figure 5C), which can be explained by the slower movement of bubbles [5] and the slower drainage process of the liquid thin film (between bubbles) [28], delaying (or diminishing) bubble coalescence. To summarize, the continuous phase properties (namely, protein concentration and phase viscosity) show vastly different influence on either bubble formation or coalescence: (1) they can control the size at bubble formation ($D_{0}$) only to a very limited extent, with $Q_{c}$ the dominating factor; (2) they significantly suppress coalescence and thus the formation of larger bubbles ($D_{coal}$).

The coefficient of variation (CV) is used to characterize the bubble size distribution (see Figure 5D–F). The CV is always higher for the coalesced bubbles than for the initially formed bubbles. The CV can only be reduced to a limited extent when we increase the continuous phase flow rate, and we obtain a minimum CV of 20% and 34% for $D_{0}$ and $D_{coal}$, respectively. The CV does decrease strongly down to 14% and 17%, respectively, when measures are taken to enhance stabilization of the freshly-created interfaces, namely by raising the protein concentration, or increasing the continuous phase viscosity. In both cases a smaller average bubble size is accompanied by a smaller CV as the bubble size distribution is narrowed on the upper end. These results indicate that to tightly control the properties of bubbles in the end product, irrespective of membrane properties, it is crucial to manipulate the operation conditions and the fluid properties to control bubble formation and, especially, to prevent bubble coalescence by sufficiently fast stabilization of the freshly-created interface and by slowing down the approaching bubbles, and thus the film drainage process.

### 3.4. Bubble Formation Dynamics—Timescales

**Figure 6:**
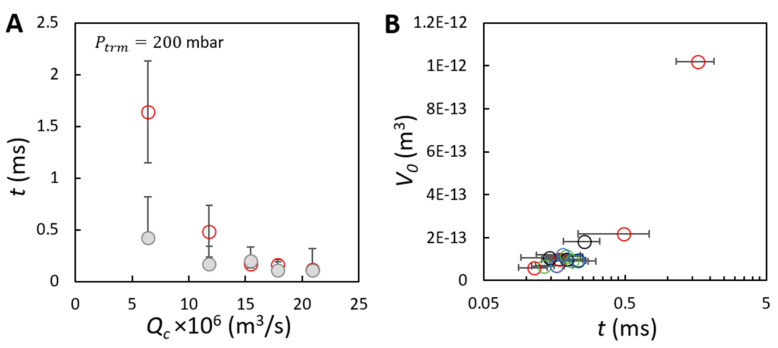
Two timescales for bubble formation at the membrane surface. (**A**) Bubble growing time (O) and the interval time (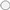) as a function of the continuous phase flow rate. (**B**) Average volume of initial bubbles as a function of the bubble growing time for the effects of $Q_{c}$ (O), $P_{trm}$ (O), c (O) and η (O). The bubble volumes correspond to that presented in Figure 4A–C. The two timescales are derived based on 30 bubble formation events, focusing on bubble formation from three positions in the field of view of the membrane in the video recorded at a frame rate of 20,000 frames per second.

Within our experimental resolution, we observe that bubble formation can be separated into two stages: a bubble growing stage and an interval stage. The latter stage corresponds to a period of time between the moment that one bubble detaches and that the next bubble appears. The two corresponding timescales are the bubble growing time (red symbols in Figure 6A) and the interval time (grey symbols in Figure 6A). For a given transmembrane pressure, the bubble growing time significantly decreases from 1.64 to 0.13 ms as $Q_{c}$ increases (Figure 6A). This is because with increasing $Q_{c}$ the shear force increases, and the bubble is snapped off faster. The corresponding interval time only slightly decreases and converges towards the bubble growing time for $Q_{c}>1.5\times 10^{-5}\;m^{3}/s$. We highlight that both timescales can decrease to hundreds of microseconds. Muijlwijk and co-authors (2017) [29] reported that in a 1 wt % *β*-lactoglobulin system, which is the main component of whey protein, bubbles are stable against coalescence only if a 100-millisecond duration (within the experimental resolution) is allowed for protein adsorption before bubble–bubble interactions occur. Additionally, in a 5 wt % whey protein system, we recently demonstrated that micrometer-sized bubbles can be sufficiently stabilized at a timescale larger than 1 millisecond; and at a timescale of ~0.01–1 millisecond, bubble formation co-exists with finite bubble coalescence [30]. Therefore, the similar observations of bubble formation and controllable bubble coalescence in the current CFMF system can be explained by the intersecting timescales of bubble formation and protein adsorption [30].

The final bubble size reflects a balance between bubble formation and bubble coalescence. The probability of bubble coalescence is steeply decreasing when a monolayer surface coverage is achieved. When the protein concentration or the continuous phase viscosity is manipulated, and both $Q_{c}$ and $P_{trm}$ are fixed, bubbles are likely to grow at a fixed surface expansion rate within a fixed period of time, ranging mostly from 0.13 up to 0.23 ms (see Figure 6B). To explain bubble stabilization within the 2.5 wt % whey protein system, we introduce a dimensionless *Péclet* number (Pé, defined as $Pé=\frac{Lv_{c}}{D}$, where D is the diffusion coefficient and L is a characteristic length), which describes the relative importance of convection and diffusion during transport of proteins. We first calculated the diffusion coefficient of *β*-lactoglobulin (as the representative component of whey protein) using the Stoke–Einstein equation and then obtained Pé≫1 (S7). Therefore, the above-mentioned surprisingly high bubble stability can be ascribed to high bulk concentration and bulk convection (enhanced mass transport of proteins). Additionally, given the bubble size encountered in this study, the highly curved surface can also accelerate the protein adsorption process and thus contribute to the high bubble stability [31]. 

## 4. Conclusions

In the cross-flow membrane foaming (CFMF) system, bubble formation is studied using whey protein as the surfactant. At a (sub)millisecond timescale, bubbles continuously grow at the pores and snap off based on a force balance mechanism. The snap-off bubble size ($D_{0}$) is dominated by the continuous phase shear force; there is almost no dependency on the transmembrane pressure. The final bubble size ($D_{coal}$) is also determined by bubble coalescence, and it is greatly reduced at higher protein concentration and/or higher continuous phase viscosity. The $D_{coal}$ can be almost equal to $D_{0}$, and the coefficient of variation (CV) can be well controlled below 20%. This means that in an ideal CFMF system where bubbles can be directly stabilized, a foam product composed of monodisperse bubbles with targeted size can be produced. 

To further understand and optimize the CFMF system, several aspects such as membrane properties and dimensionless analysis of membrane foaming systems, etc., can be investigated in-depth in a future work. For example, as was reported for membrane emulsification, specifically small membrane pores allow the formation of monodisperse and small droplets, which have a higher storage stability; meanwhile, smaller pore size may screen out the effects of other tested parameters during emulsification [11]. Hence, it is also interesting to explore the effect of the small pore size and unravel the potential competitive effects of small pore size and continuous phase shear on the formation mechanism and the probability of coalescence of bubbles. This could either be studied for a range of membranes with a well-defined pore size distribution, or, to gain insight on the individual pore level, using microfluidic devices operating under similar shear conditions.

In general, our study provides insights into what happens during the production of (food) foams at a (sub)millisecond timescale, and the insights generated can also be used to understand and steer large-scale processes better.

## Data Availability

Not applicable.

## Supplementary Materials

The following are available online at https://www.mdpi.com/article/10.3390/membranes11090710/s1, Figure S1: Protein concentration as a function of times of re-use. The initial concentration is 1 wt %,  Figure S2. Bubble coalescence observed at two distinct positions in the CFMF system. **A.** Bubble diameter as a function of the transmembrane pressure, for $Q_{c}=1.1\times 10^{-5}{\;m}^{3}/s$ in a 1 wt %  whey protein system. **B.** Number of coalesced bubbles. In A, the bubble size $D_{0}$ measured in the membrane module is represented by un-filled circle (O). In A and B, the bubble size $D_{coal}$ is indicated by filled circles and the number of coalesced bubbles is indicated by filled triangles, obtained at 10 cm (, ) and 100 cm (, ) downstream of the module. The *CV* for $D_{coal}$ measured at the two positions vary in the range of 26~35%, Figure S3. Bubble size versus the shear force ($v_{c}\eta$). These data are shown in the manuscript in Figure 4A (for $P_{trm}=$ 200 mbar) and C. The fitting was performed by non-linear least square regression, Figure S4-1. Schematic overview of potential variation in transmembrane pressure along the length of the membrane inside the module. The schematic is not drawn to scale, Figure S4-2. Histogram plots for bubbles with $D_{0}$ (grey line) and $D_{coal}$ (red line). The corresponding experimental conditions for A-C are marked in the figures, Figure S5. Number of coalescence events. In the order of A-C, the effects of the continuous phase flow rate (for a series of transmembrane pressures), protein concentration and the continuous phase viscosity are presented, Figure S6. Bubble diameters as a function of transmembrane pressure. The continuous phase flow rate is fixed at $Q_{c}=1.5\times 10^{-5}{\;m}^{3}/s$. For comparison, the initial bubbles with $D_{0}$ are only shown for 1 wt % whey protein (O), and the $D_{coal}$ is evaluated for both 1 wt % () and 2.5 wt % () whey protein solutions, S7. Calculation of D and $P_{é}$.
