# Supplementary material for: Mapping Bubble Formation and Coalescence in a Tubular Cross-Flow Membrane Foaming System
Source: Membranes (Basel). 2021 Sep 15;11(9):710. doi: 10.3390/membranes11090710 (PMC8468550; doi:10.3390/membranes11090710)
Supplement: Supplementary file 1 [file membranes-11-00710-s001.zip › membranes-1347569-supplementary.pdf]

membranes

Supplementary Materials

Mapping Bubble Formation and Coalescence in a Tubular Cross-Flow Membrane Foaming System

Boxin Deng, Tessa Neef, Karin Schroën and Jolet de Ruiter *

Food Process Engineering Group, Department of Agrotechnology & Food Science, Wageningen University, Bornse Weilanden 9, 6708 WG Wageningen, The Netherlands; boxin.deng@wur.nl (B.D.); tessa.neef@wur.nl (T.N.); karin.schroen@wur.nl (K.S.)
* Correspondence: jolet.deruiter@wur.nl

S1 Protein concentration loss over time

Due to the high applied continuous phase flow rate, a 4 L protein solution can run out within 3 min in the current experimental set-up. Hence, re-use of the protein solutions is considered to be more practically efficient and sustainable. Before doing that, to figure out whether the potential decrease in protein concentration may influence the experimental results, the concentration of the protein solutions taken from the feed tank (for the continuous phase) after re-use of 0, 1, 3, 5, 7 and 9 times, was measured in triplicate using the Dumas method. The results indicate that after 9 times of re-use, the protein concentration only decreases by approximately 20%, which can be ignored (Figure S1). Hence, the protein solution was re-used for up to 10 times.

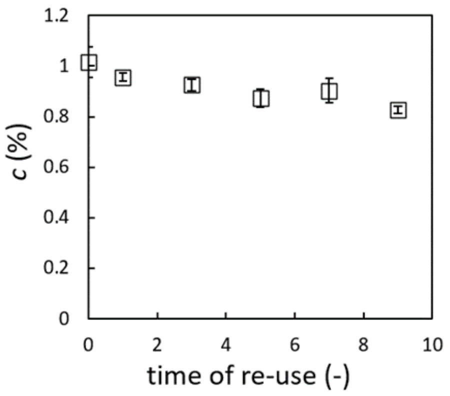

Figure S1. Protein concentration as function of times of re-use. The initial concentration is 1 wt %.

S2 Comparison of bubble coalescence at two distinct positions downstream the module

The flow cell is attached to the module downstream at a distance of either 10 cm or 100 cm. The D_{coal} values obtained for each scenario are compared to confirm whether coalescence mainly occurs in the membrane module during or shortly after bubble formation (10 cm), or that bubbles continue to coalescence along the flow path (100 cm). In Figure S2, similar D_{coal} values (and thus similar N values) show that coalescence mainly happens in the membrane module in the current system.

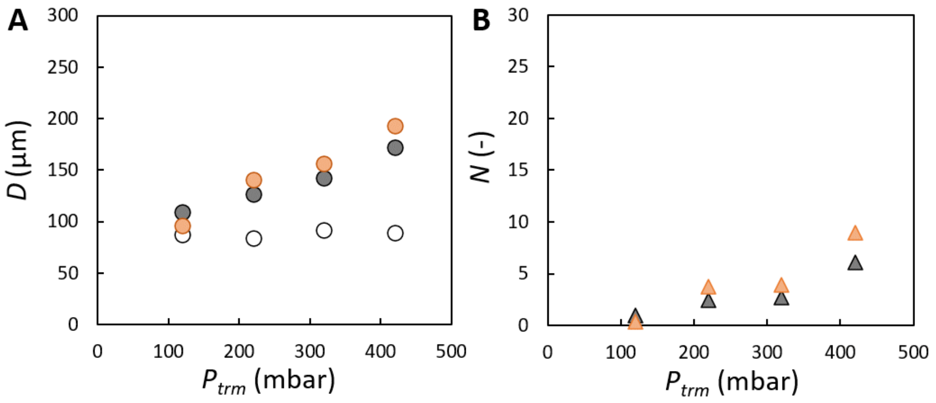

Figure S2. Bubble coalescence observed at two distinct positions in the CFMF system. A. Bubble diameter as a function of the transmembrane pressure, for $Q_c = 1.1 \times 10^{-5}$ m^3/s in a 1 wt % whey protein system. B. Number of coalescence events. In A, the bubble size D_0 measured in the membrane module is represented by un-filled circle (O). In A and B, the bubble size D_{coal} is indicated by filled circles and the number of coalesced bubbles is indicated by filled triangles, obtained at 10 cm (●, ▲) and 100 cm (●, ▲) downstream of the module. The CV for D_{coal} measured at the two positions vary in the range of 26% ~ 35%.

S3 Fitting of the scaling ~ Ca^{-n}

Based on the bubble formation mechanism in the CFMF system, we proposed a scaling with Ca^{-n}, where $Ca = \frac{v_c\eta}{\gamma}$ is the dimensionless capillary number, to describe the bubble size (D_0) for the effects of continuous phase velocity and viscosity. Assuming a constant surface tension, we fitted the proposed scaling (which is simplified as $(v_c\eta)^{-n}$) to the experimental data and obtained the power-law exponent $n = 0.8 \pm 0.1$. Both the experimental data and fitted scaling are shown in Figure S3.

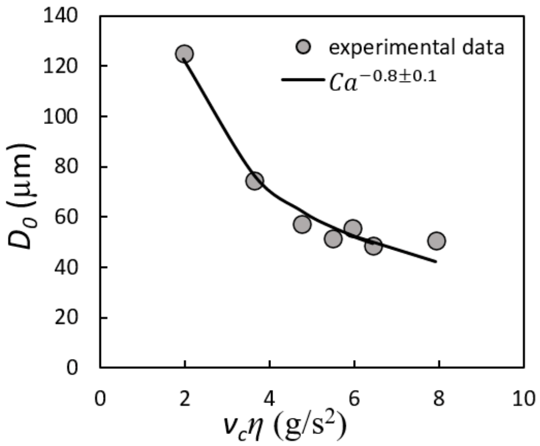

Figure S3. Bubble size versus the shear force ($v_c\eta$). These data are shown in the manuscript in Figure 4A (for P_{trm} ==200 mbar) and C. The fitting was performed by non-linear least square regression.

S4 General bubble behavior in the CFMF system – Pressure analysis

Ideally, bubbles growing at the membrane surface should be subjected to the same experimental conditions (once a certain series of experimental conditions are applied), and should show no dependency on the position of the membrane (e.g. positioning upstream or downstream inside the module).

However, the continuous phase pressure may drop along the length of the membrane in the direction of flow. Consequently, at a certain applied pressure conditions (P_d, P_c), an increasing transmembrane pressure can be expected to act across the pores along the membrane: P_{trm} at the beginning of the module and $P_{trm}{}'$ at the outlet of the module (see the sketch in Figure S4-1, $P_{trm} < P_{trm}{}'$). The variation of D_0 with P_{trm} is relatively

minor for $P_{trm} = 100 - 300$ mbar (see Figure 3B), but if the pressure increases towards 400 mbar (or even higher) along the membrane, at that point, slightly larger bubbles could form directly from the pores.

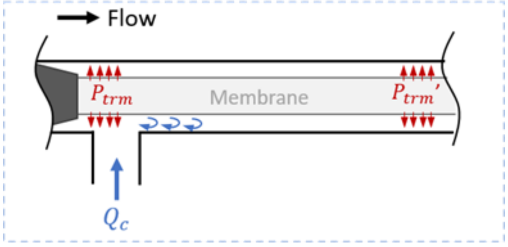

Figure S4-1. Schematic overview of potential variation in transmembrane pressure along the length of the membrane inside the module. The schematic is not drawn to scale.

The observations in the membrane module can be made with only two narrow strips along the membrane in the focal plane (see Figure S4-1). For the analysis we chose bubbles formed upstream of the membrane across from the continuous phase inlet, which experiences laminar flow conditions. In contrast, we have observed that near the continuous phase inlet bubbles are formed in a less-predictable fashion (in terms of bubble size, formation frequency and coalescence), due to the presence of circulating flows (indicated by blue eddies, Figure S4-1). Specifically, relatively larger bubbles have been observed to form directly at the membrane surface. The presence of these bubbles in the module means that the reported D_0 (from exclusively the strip located across from the continuous phase inlet) may be a slight underestimation of the bubble diameter averaged over the full membrane, and thus the number of coalescence events will be overestimated. In addition, bubbles that are flowing near the continuous phase inlet are stretched (and deformed) within the circulating flow and are occasionally observed to break up into (two) smaller bubbles.

Even if we measure D_0 *locally* directly upstream of the membrane across the continuous phase inlet, the measurement seems to be still representative for the full membrane. The size distribution of D_0 (grey line in Figure S4-2) shows good similarity with D_{coal} that includes bubbles formed *anywhere* along the membrane (red line); that is, for conditions that would largely limit coalescence (namely, at high protein concentration or a high continuous phase viscosity, Figure S4-2 B,C). The larger difference observed between D_0 and D_{coal} as shown in Figure S4-2 A can then be largely ascribed to the high extent of bubble coalescence.

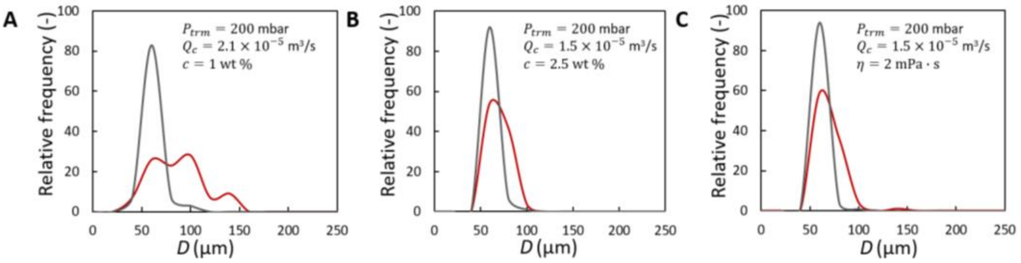

Figure S4-2. Histogram plots for bubbles with D_0 (grey line) and D_{coal} (red line). The corresponding experimental conditions for A-C are marked in the figures.

S5 The number of coalescence events, N

To illustrate the extent of coalescence, the number ($N = \frac{V_{coal}}{V_0} - 1$) of coalescence events is estimated. The V_0 and V_{coal} are the volumes of bubbles with D_0 and D_{coal}, which are shown in Figure 4 and 5, respectively, in the manuscript. Generally, bubbles

increasingly coalesce as Q_c and P_{trm} increase, while the coalescence can be suppressed with increasing protein concentration and the continuous phase viscosity.

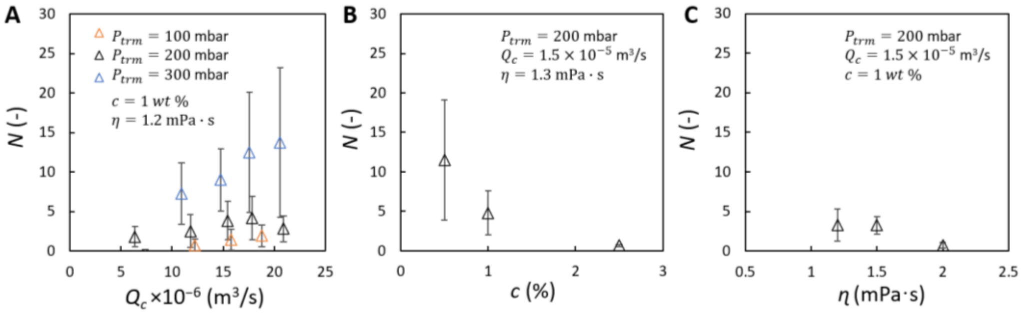

Figure S5. Number of coalescence events. In the order of A-C, the effects of the continuous phase flow rate (for a series of transmembrane pressures), protein concentration and the continuous phase viscosity are presented.

S6 Bubble coalescence can be suppressed with 2.5 wt % whey protein

To demonstrate the stabilizing effect of 2.5 wt % whey protein on D_0, the D_{coal} is evaluated as a function of P_{trm} and compared with the 1 wt % whey protein system. For the 2.5 wt % whey protein system, similar D_0 and D_{coal} can be obtained, showing no dependency on $P_{trm.}$; thus showing that coalescence is effectively suppressed.

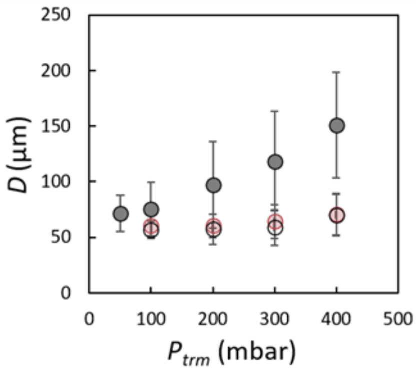

Figure S6. Bubble diameters as a function of transmembrane pressure. The continuous phase flow rate is fixed at $Q_c = 1.5 \times 10^{-5}$ m^3/s. For comparison, the initial bubbles with D_0 are only shown for 1 wt % whey protein (O), and the D_{coal} is evaluated for both 1 wt % (●) and 2.5 wt % (O) whey protein solutions.

S7 Calculation of D and Pé

The diffusion coefficient is calculated using the Stokes-Einstein equation $D = \frac{kT}{6\pi\eta r}$ with $k = 1.38 \times 10^{-23}\,\mathrm{J\,K^{-1}}$, $T = 293\,\mathrm{K}$, $\eta = 1.3 \times 10^{-3}\,\mathrm{kg\,m^{-1}s^{-1}}$ and $r = 0.066M^{1/3} \times 10^{-9}$ [1]. The last item is the minimum molecule radius (in meter, m) which is expressed as function of molecular weight (M, in Daltons) with M for β-lactoglobulin being 18000 Da.

The Péclet number is calculated as $Pe = \frac{v_c L}{D}$. The characteristic length scale (L) in the CFMF system is set as $5.5 \times 10^{-4}\ m$ which represents the gap distance between the membrane surface and the inner wall of the membrane module. The velocity (v_c) of the continuous phase flowing through the gap is estimated as $4\ m/s$. The estimate for the Péclet number based on the diffusion coefficient of β-lactoglobulin is $2 \times 10^7 \gg 1$, indicating the dominance of bulk convection (over bulk diffusion) and thus the enhanced mass transport of proteins, leading to improved coalescence stability of the bubbles.

References

1. Erickson, H.P. Size and Shape of Protein Molecules at the Nanometer Level Determined by Sedimentation, Gel Filtration, and Electron Microscopy. *Biol. Proced. Online* **2009**, *11*, 32–51, doi:10.1007/s12575-009-9008-x.
2. Zanatta, V.; Rezzadori, K.; Penha, F.M.; Zin, G.; Lemos-Senna, E.; Petrus, J.C.C.; Di Luccio, M. Stability of oil-in-water emulsions produced by membrane emulsification with microporous ceramic membranes. *J. Food Eng.* **2017**, *195*, 73–84, doi:10.1016/j.jfoodeng.2016.09.025.
3. Kobayashi, I.; Yasuno, M.; Iwamoto, S.; Shono, A.; Satoh, K.; Nakajima, M. Microscopic observation of emulsion droplet formation from a polycarbonate membrane. *Colloids Surfaces A: Physicochem. Eng. Asp.* **2002**, *207*, 185–196, doi:10.1016/s0927-7757(02)00093-6.
4. Piacentini, E.; Imbrogno, A.; Drioli, E.; Giorno, L. Membranes with tailored wettability properties for the generation of uniform emulsion droplets with high efficiency. *J. Membr. Sci.* **2014**, *459*, 96–103, doi:10.1016/j.memsci.2014.01.075.
5. van der Graaf, S.; Steegmans, M.; van der Sman, R.; Schroen, K.; Boom, R. Droplet formation in a T-shaped microchannel junction: A model system for membrane emulsification. *Colloids Surfaces A: Physicochem. Eng. Asp.* **2005**, *266*, 106–116, doi:10.1016/j.colsurfa.2005.06.019.
